# Low grade of osteoarthritis development after Latarjet procedure with a minimum 5 years of follow-up: a systematic review and pooled analysis

**DOI:** 10.1007/s00167-021-06771-w

**Published:** 2021-10-22

**Authors:** Alessandra Menon, Chiara Fossati, Mauro Magnani, Sara Boveri, Riccardo Compagnoni, Pietro Simone Randelli

**Affiliations:** 1 Laboratory of Applied Biomechanics, Department of Biomedical Sciences for Health, Università degli Studi di Milano, Via Mangiagalli 31, 20133 Milan, Italy; 2 U.O.C. 1a Clinica Ortopedica, ASST Centro Specialistico Ortopedico Traumatologico Gaetano Pini-CTO, Piazza Cardinal Ferrari 1, 20122 Milan, Italy; 3 Research Center for Adult and Pediatric Rheumatic Diseases (RECAP-RD), Department of Biomedical Sciences for Health, Università degli Studi di Milano, Via Mangiagalli 31, 20133 Milan, Italy; 4 Scientific Directorate, IRCCS Policlinico San Donato, Piazza Malan 1, 20097 Milan, Italy

**Keywords:** Glenohumeral arthritis, Shoulder arthropathy, Latarjet procedure, Shoulder instability, Dislocation arthropathy, Shoulder osteoarthritis

## Abstract

**Purpose:**

The purpose of this systematic review and pooled analysis was to evaluate incidence and risk factors for glenohumeral osteoarthritis (OA) in patients who underwent Latarjet procedure with a minimum of 5-year follow-up.

**Methods:**

The PRISMA guidelines were followed to perform this systematic review. PubMed and EMBASE were searched up to February 29, 2020 for English, human in vivo studies that evaluated glenohumeral OA in patients undergoing Latarjet procedure at least 5 years after surgery. A pooled analysis on the included databases sent by authors was performed to evaluate the risk factors influencing the development or progression of dislocation arthropathy after the Latarjet procedure.

**Results:**

Four studies, including a total of 280 patients (213 males and 67 females), were analysed. In our study population, the median age at surgery was 25.0 years (range 20.8–32.6 years). and 92.1% were athletes. In 90% of the cases, the number of dislocations before surgery were fewer than 5. The recurrence of instability after Latarjet procedure was observed only in seven patients (2.5%). The position of the bone graft resulted flush to the anterior glenoid rim in 238 cases (85.3%), medial in 8 (2.9%) and overhanging in 33 (11.8%). Radiological signs of development or progression of shoulder OA were observed in 25.8% of the patients, of which 88.6% presented a grade 1 of OA according to Samilson and Prieto classification. The overhanging position of the bone graft resulted statistically significant for onset or worsening of OA. The age at surgery, the number of dislocations before surgery and the Hill–Sachs lesion were not significantly associated with joint degeneration. Instead, hyperlaxity showed a prevention role in the development of OA after open Latarjet procedure.

**Conclusion:**

The Latarjet procedure is a valid and safe surgical treatment in recurrent anterior shoulder instability with a low risk of developing moderate or severe OA also at long-term follow-up. The overhanging position of the bone graft represents the principal risk factor of joint degeneration, whereas the hyperlaxity seems to be protective. Finally, age, gender, time between first dislocation and surgery, and number of dislocations do not seem to affect the onset of OA after Latarjet procedure. Therefore, an accurate execution of the Latarjet procedure can be considered a valid treatment even in young and athletes thanks to the low recurrence rates and the low development of major long-term complications.

**Level of evidence:**

IV.

**Supplementary Information:**

The online version contains supplementary material available at 10.1007/s00167-021-06771-w.

## Introduction

Traumatic anterior shoulder dislocation is a common pathology that usually affects competitive-level sporting males between 20 and 30 years old [1, 27], with a reported incidence of 24 per 100.000 persons/year [39, 63]. Glenoid and humeral bony defects, in association with soft tissue injuries, often lead to recurrent shoulder instability [12, 15, 29, 57].

The most reliable surgical approach to anterior shoulder instability remains challenging [5, 61]. In clinical practice, anatomical surgeries, such as arthroscopic Bankart repair, represent the best surgical options in patients with no or slight bone loss. The non-anatomical techniques, such as the Latarjet procedure, are preferred in case of glenoid bone loss > 25% (inverted pear glenoid), significant anteroinferior capsular deficit, patients involved in competitive or contact sports (e.g., rugby), and failed stabilization procedures [7].

In the Latarjet procedure, described for the first time in 1954 [37], the coracoid process along the conjoint tendon is distally transferred and screw fixed to the anterior border of the glenoid. This technique provides shoulder stability with a triple mechanism: restoration of the bone loss provided by the coracoid process, the sling effect created by the dynamic tension on the inferior part of the subscapularis exerted by the conjoint tendon, and, finally, the Bankart effect of capsulolabral repair [45, 62].

Despite excellent long-term results in terms of recurrence rate, the Latarjet procedure shows some complications, including graft malposition and non-union, nerve and vascular injury, hematoma, infection, loss of external rotation, and development of osteoarthritis (OA) [17].

The glenohumeral OA is one of the most feared long-term complications after Latarjet procedure in particular because the patients who underwent this surgery are on average young. Several factors, including advanced age at the first dislocation [36], high number of pre-operative dislocations, contact sports (e.g., rugby and basketball) or heavy manual works (e.g., carpentry and bricklayer), glenoid or humeral head fractures, rotator cuff tears, high-energy trauma and technical errors as bad graft positioning, intra-articular hardware and excessive capsular tightening, have been suggested to increase the risk of OA in patients who underwent the Latarjet procedure [36, 43]. However, studies with limited evidence and small samples do not allow a definitive conclusion.

The purpose of this study was to perform a systematic review of the literature and conduct the first pooled analysis to establish the incidence and risk factors for long-term glenohumeral OA after a Latarjet procedure in patients with a minimum follow-up of 5 years.

## Materials and methods

**Fig. 1:**
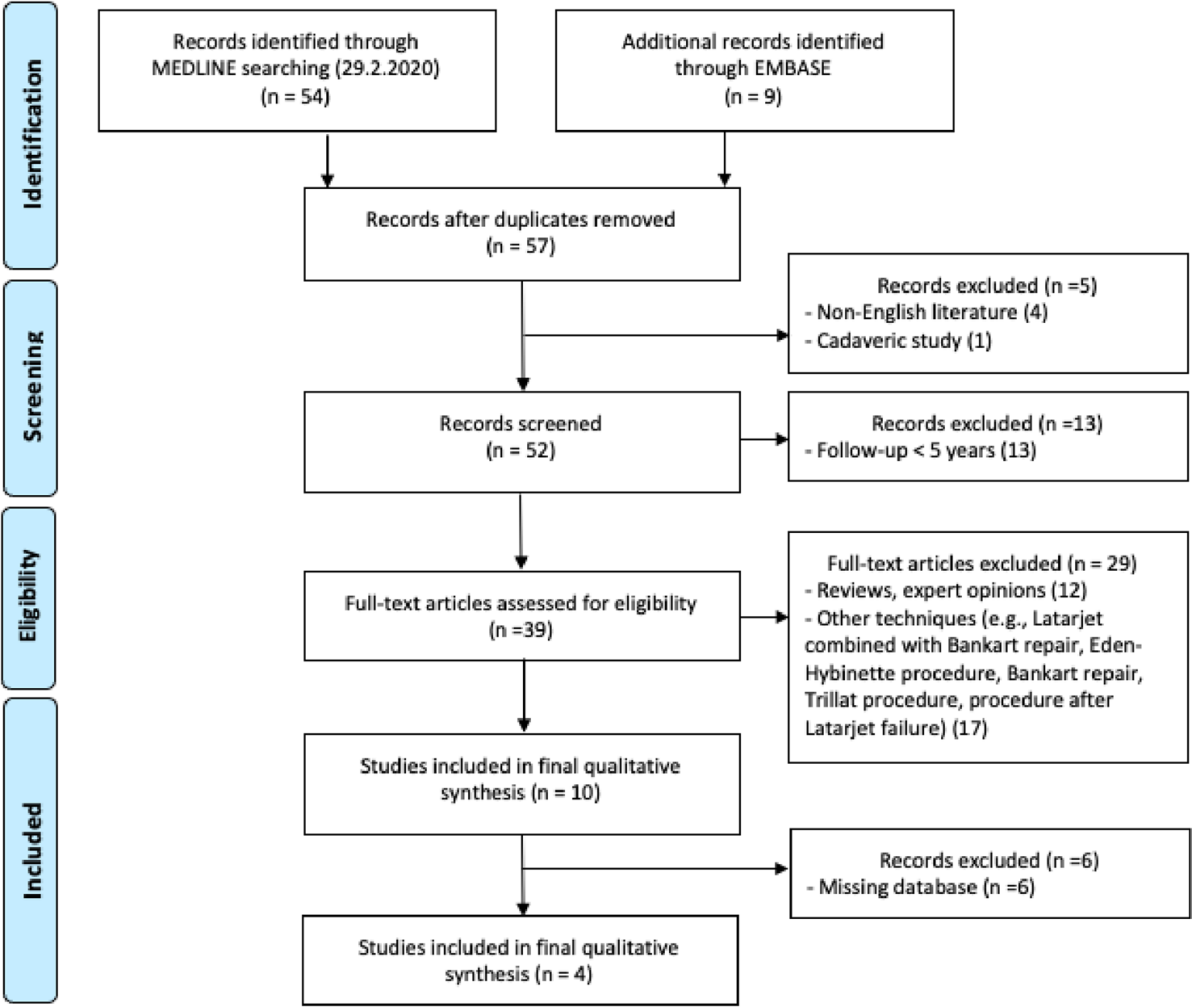
Flowchart of the study selection

A systematic review to identify all studies reporting glenohumeral OA after Latarjet procedure at a minimum follow-up of 5 years was performed. The PRISMA guidelines (Preferred Reporting Items for Systematic Reviews and Meta-Analyses) were followed to perform this systematic review of the literature and to present the results [44]. A protocol was written stating the purpose of the review and the search strategy and registered on PROSPERO (registration ID: CRD42021089462). A flow diagram according to PRISMA guidelines summarizes our selection protocol (Fig. 1).

### Primary and secondary outcomes

Primary and secondary outcomes were planned a priori. The main outcome was the incidence of glenohumeral OA in patients who underwent Latarjet procedure with a minimum of 5 years follow-up. The secondary outcomes included the assessment of the risk factors in developing glenohumeral OA after this surgery.

### Search strategy and selection criteria

An electronic search of the literature was performed in the MEDLINE database via PubMed and Embase database from the databases’ inception up to February 29, 2020, using the following search string for title and abstract: (Latarjet OR Coracoid Bone Block) AND {[(Dislocation Arthropathy) OR (Arthritis)] OR [(Arthrosis) OR (Degenerative Changes)]}. MeSH terms were used only for “Arthritis”.

The search was limited to English literature. Meta-analyses, systematic and narrative reviews, and non-human studies (animal and cadaver studies) were excluded. The search strategy for each specific search is shown in Appendix A.

### Study selection

According to the methodology recommended by Harris et al. [23], after the deletion of duplicates, the title and abstract of all identified studies were independently examined by two reviewers (C.F., M.M.), who applied the study eligibility criteria. When the title and abstract were pertinent, the article was analysed.

Inclusion criteria were studies in the English that evaluated glenohumeral OA in patients undergoing Latarjet procedure at least 5 years after surgery.

Exclusion criteria were studies not meeting inclusion criteria, studies using different techniques/procedures to manage glenohumeral OA, narrative and systematic reviews, meta-analyses and cadaveric studies.

In case of disagreement between reviewers, researchers obtained the consensus discussing the full text. In case of persistent disagreement, a third reviewer (A.M.) was consulted.

Finally, eligible articles underwent a full-text review for a more detailed evaluation. Both reviewers also manually cross-referenced to ensure that all potential studies were included. Reviewers were not blinded to the authors or affiliated institutions of the retrieved studies. The final list of included studies was agreed to by consensus, and their authors were contacted by email to obtain the raw data. A pooled analysis on the included databases sent by authors was performed to evaluate the risk factors influencing the development or progression of dislocation arthropathy after the Latarjet procedure.

### Risk of bias assessment

Two reviewers (A.M., M.M.) independently assessed methodological quality of the included studies according to the MINORS (Methodological Index for Nonrandomized Studies) checklist [53]. On the base of this tool, 8 items for non-comparative studies and 12 items for comparative studies have been evaluated with a score that varies from 0 to 2 (0: not reported; 1: reported but inadequate; 2: reported and adequate). Therefore, the maximum global score was 16 for a non-comparative study and 24 for a comparative study.

The level of evidence of each article was assessed using the 2003 Journal of Bone and Joint Surgery definitions for orthopaedic publications [60].

### Data extraction

Two reviewers (A.M. and C.F.) independently extracted study data using a standardized data extraction form predefined according to the protocol. Discordance was resolved by both reviewers rechecking their extracted data until data sheets corresponded. If no consensus could be reached, a third reviewer (M.M.) was consulted. Authors extracted information regarding the characteristics of the studies (author, year and journal of publication, study design and level of evidence, number of patients and shoulders) and participants (sex, age at surgery and follow-up, dominant shoulder or not, follow-up duration, hyperlaxity, sport and physical activity, number of pre-operative dislocations and subluxations, pre-operative validated outcome measures, pre-operative OA according to Samilson and Prieto, pre-operative glenoid bone loss/glenoid defect, pre-operative Hill-Sachs lesion, position of graft, graft healing alteration, screw problems), and the clinical, functional and radiographic outcomes of the treatment at the final follow-up (OA according to Samilson and Prieto, validated outcome measures, sport, and physical activity, recurrence of instability, complications and adverse events, recurrence of post-revision instability, number of re-dislocations, satisfaction). Where possible, the compiled data from individual studies were pooled together.

### Statistical analysis

The original datasets of the studies, for which the authors provided us with databases, were analysed. We considered data of survival times subject to right-censoring, for individuals the true survival times exceeded the censoring time. Until the event of interest was not observed precisely, the database had only one follow-up visit after surgery. The approach for analysing interval-censored survival data was the use of nonparametric estimation of the survival function.

The proportional hazards regression model for the interval-censored current time to event data was used to estimate univariate and multivariate hazard ratios (HR) with/and their 95% confidence intervals (95% CI) for evaluating the association between covariates and post-surgery osteoarthritis outcome.

For all analyses, the significance level was set at a *p* value lower than 0.05.

Statistical analysis was performed using SAS software, version 9.4 (SAS Institute, Inc., Cary, NC).

## Results

### Study selection and study characteristics

As shown in Fig. 1, a total of 63 references were identified (54 from MEDLINE and 9 from EMBASE). After eliminating duplicates and screening titles and abstracts, 39 were assessed for eligibility. Of these 39 studies, 29 were excluded [3, 4, 8–10, 14, 16, 17, 19, 21, 26, 28, 30–35, 38, 40–42, 46–48, 54, 55, 58, 59]. Ten studies were included in final qualitative synthesis and, finally, six of which [2, 18, 20, 24, 25, 52] were not taken into consideration because the databases were not available. Therefore, 4 studies [11, 36, 43, 45], which included 280 patients who underwent the Latarjet procedure, were analysed. All these analysed studies were retrospective. The mean follow-up was more than 5 years in each of them. The mean MINORS score was 9.5 out of a possible 16 for non-comparative studies (Table 1).

**Table 1:** Study characteristics

Study	Year	Design	Level of evidence	MINORS score
Bouju et al. [11]	2014	Retrospective	IV	8/16
Lädermann et al. [36]	2013	Retrospective	III	10/16
Mizuno et al. [43]	2014	Retrospective	IV	10/16
Neyton et al. [45]	2012	Retrospective	IV	10/16

*MINORS* Methodological Index for Nonrandomized Studies

The study population was characterized by general information and clinical data (Table 2). Three out of four studies have even described hyperlaxity [11, 36, 43] and the Hill–Sachs lesion [11, 43, 45].

**Table 2:** Characteristics of the study population

	Overall (*N* = 280)	Bouju (*N* = 58)	Ladermann (*N* = 117)	Mizuno (*N* = 68)	Neyton (*N* = 37)
Age at surgery in years (95% CI)	25.0 (20.8–32.6)	24.4 (20.2–35.6)	26.6 (21.9–33.7)	24.0 (21.0–37.0)	23.0 (20.0–27.0)
Age at follow-up in years (95% CI)	42.0 (37.0–49.4)	38.0 (32.5–46.0)	44.0 (39.0–52.0)	45.0 (40.0–54.0)	34.0 (30.0–39.0)
Length of follow-up in months (95% CI)	210.8 (199.2–225.1)	152.4 (141.1–167.8)	214.7 (199.7–243.4)	243.2 (237.3–257.7)	174.5 (123.9–196.0)
Gender (%)					
Male	213 (76.1)	40 (69.0)	82 (70.1)	54 (79.4)	37 (100)
Female	67 (23.9)	18 (31.0)	35 (29.9)	14 (20.6)	–
Dominancy (%)					
Left	30 (12.6)	5 (16.7)	15 (13.4)	10 (14.7)	–
Right	208 (87.4)	53 (91.4)	97 (86.6)	58 (85.3)	–
Dominancy surgery (%)					
Operated dominant arm	136 (57.1)	39 (67.2)	58 (51.8)	39 (57.4)	–
No operated dominant arm	102 (42.9)	19 (32.8)	54 (48.2)	29 (42.6)	–
Sport pre-op (%)					
Yes	258 (92.1)	58 (100)	101 (86.3)	62 (91.2)	37 (100)
No	22 (7.8)	–	16 (13.7)	6 (8.8)	–
Hyperlaxity (%)					
Yes	37 (15.4)	13 (22.4)	20 (17.5)	4 (5.9)	–
No	203 (84.6)	45 (77.6)	94 (82.5)	64 (94.1)	–
Recurrence of instab (%)					
Yes	7 (2.5)	1 (1.7)	2 (1.7)	4 (5.9)	0
No	273 (97.5)	57 (98.3)	115 (98.3)	64 (94.1)	37 (100)
OA pre-op (%)					
Yes	16 (5.9)	2 (3.5)	3 (2.7)	8 (11.8)	3 (8.1)
No	257 (94.1)	56 (96.5)	107 (97.3)	60 (88.2)	34 (91.9)
Grade Samilson and Prieto pre-op (%)					
> 1	23 (8.2)	2 (3.5)	10 (8.6)	8 (11.8)	3 (8.1)
0	257 (91.8)	56 (96.6)	107 (91.5)	60 (88.2)	34 (91.9)
Glenoid bone loss (%)					
1	67 (41.1)	20 (34.5)	–	20 (29.4)	27 (73.0)
0	96 (58.9)	38 (65.5)	–	48 (70.6)	10 (27.0)
Graft healing (%)					
1	26 (9.3)	14 (24.1)	7 (6.0)	1 (1.5)	4 (11.1)
0	254 (90.7)	44 (75.0)	110 (94.0)	67 (98.5)	32 (88.9)
Hill–Sachs (%)					
No or small	53 (32.5)	29 (50.0)	–	12 (17.6)	12 (32.4)
Mild or large	110 (67.5)	29 (50.0)	–	56 (83.4)	25 (67.6)
Number of dislocations (%)					
≤ 5	252 (90.0)	48 (82.9)	117 (100)	50 (73.5)	37 (100)
> 5	28 (10.0)	10 (17.1)	–	18 (26.5)	–
Number of subluxations (%)					
≤ 5	268 (95.7)	56 (96.6)	117 (100)	58 (85.3)	37 (100)
> 5	12 (4.3)	2 (3.4)	–	10 (14.7)	–
Position of graft coracoid (%)					
Flush	238 (85.3)	51 (87.9)	99 (85.3)	53 (77.9)	35 (94.6)
Medial	8 (2.9)	–	–	7 (10.3)	1 (2.7)
Over	33 (11.8)	7 (12.1)	17 (14.7)	8 (11.8)	1 (2.7)
Delta of grade Samilson and Prieto (%)					
No difference pre- and post-op (grade 0)	194 (71.3)	53 (91.4)	68 (61.8)	48 (70.6)	25 (69.4)
No difference pre- and post-op (grade 1)	8 (2.9)	1 (1.7)	–	4 (5.9)	3 (8.3)
Variation 0 to 1 or 2 or 3	62 (22.9)	3 (5.2)	39 (35.5)	12 (17.6)	8 (22.2)
Variation 1 to 2 or 3	8 (2.9)	1 (1.7)	3 (2.7)	4 (5.9)	–

Each author also evaluated the patients with radiographic protocols, including anteroposterior (AP) and Bernageau views [6]. The AP view was used to assess the presence of glenohumeral OA according to Samilson and Prieto’s classification [51]. The Bernageau view was performed to evaluate the position of the bone graft; three positions were described: flush when the graft and the glenoid were in line, medial when the graft was relatively medial to the joint, and, finally, overhanging when the graft was lateral compared to the glenoid joint line. A radiological assessment was performed preoperatively and at the final follow-up to evaluate the joint degeneration in all studies.

### Synthesis of results

The pooled analysis includes 280 patients, of whom 213 males and 67 females. Total characteristics of the study population are included in Table 2.

The age at surgery was not statistically significant for the appearance or worsening of OA in both univariate and multivariate (respectively, HR > 25 vs. ≤ 25 years: 1.56 (95% CI 0.98–2.49), n.s.) analysis. The same results in univariate analysis were assessed for the number of dislocations before surgery. Moreover, the Hill–Sachs lesion was not significant for the joint degeneration.

Instead, the bone graft position resulted statistically significant in univariate analysis. Compared to flush and medial positions, patients who had an overhanging position of the graft had a statistically significant higher risk of onset or worsening of OA (HR: 4.90 (95% CI 2.86–8.40), *p* < 0.001).

On the other hand, hyperlaxity showed to be able to significantly prevent OA after the Latarjet procedure (HR = 0.31 (95% CI 0.11–0.86), *p* = 0.02) (Table 2). Adjusted multivariate analysis for age at surgery and gender showed no statistical significance for hyperlaxity (HR: 0.38 (95% CI 0.13–1.10), n.s.) but maintained statistical significance for the overhanging position of the graft (HR: 4.92 (95% CI 2.75–8.79), *p* < 0.001) (Table 3).

**Table 3:** Hazard ratio (HR) and 95% confidence intervals (CI 95%) adjusted for sites

	HR (CI 95%)			
	Univariate	*p* value	Multivariate	*p* value
Age to surgery in years				
≤ 25	Ref			
> 25	1.56 (0.98–2.49)	n.s	1.56 (0.91–2.69)	n.s
Gender				
Female	Ref			
Male	1.29 (0.73–2.28)	n.s	1.19 (0.64–2.20)	n.s
Dominancy				
Left	Ref			
Right	0.78 (0.47–1.29)	n.s		
Hyperlaxity				
No	Ref			
Yes	0.31 (0.11–0.86)	0.02	0.38 (0.13–1.10)	n.s
Recurrence of instability				
No	Ref			
Yes	1.03 (0.24–4.30)	n.s		
Glenoid bone loss				
0	Ref			
1	1.95 (0.96–3.98)	n.s		
Grade healing				
0	Ref			
1	1.13 (0.48–2.67)	n.s		
Sport post-op				
No	Ref			
Yes	0.42 (0.10–1.74)	n.s		
Hill–Sachs				
No	Ref			
Yes	1.58 (0.64–3.91)	n.s		
Dominancy				
No operated	Ref			
Operated	0.78 (0.47–1.29)	n.s		
Number of dislocations				
≤ 5	Ref			
> 5	0.96 (0.38–2.43)	n.s		
Number of subluxations				
≤ 5	Ref			
> 5	1.51 (0.51–4.50)	n.s		
Position of graft				
Small/Medial	Ref			
Over	4.90 (2.86–8.40)	<0.001	4.92 (2.75–8.79)	<0.001

## Discussion

The most important finding of the present study is that the Latarjet technique exposes patients to relatively moderate risk of developing joint degeneration (25%), and only in very few patients (6.4%), the grade of OA is significant (grade 2 or 3 according to Samilson and Prieto classification). This result has a significant clinical impact, considering that this procedure is usually performed on young and active patients.

This work represents the first systematic review and pooled analysis evaluating the development of glenohumeral OA after the open Latarjet procedure at long-term follow-up. This surgical technique results in good or excellent functional outcomes, with a generally high patient satisfaction (over 90%) [18, 49] and a great rate of return to sport at preinjury level in athletes (almost 80%) [50].

The results reported in this study describe a lower incidence of OA than those previously reported by other authors [13, 52], probably because of the shorter follow-up. Hovelius et al. [24] found a mild OA in 35.1% of shoulders and a moderate or severe dislocation arthropathy in 14% at 15 years of follow-up. Singer et al. [52] in a follow-up study of 20 years, showed 29% of significant OA (grade 2 or 3 of Samilson and Prieto classification). Chillemi et al. [13], in a 24-years follow-up study, also assessed the presence of OA in 52.5% of shoulders at the final follow-up (grade 2 or 3 was found in 25% of the shoulders, according to Samilson and Prieto classification). Despite these signs of joint degeneration, all these authors described good-to-excellent results and high level of satisfaction among their patients.

A recent systematic review [28], which analysed studies with a follow-up of at least 10 years, assessed a development of OA at the final follow-up (mean 16.6 years) in 38.7% of overall shoulders (26.5% with grade 1 of Samilson and Prieto classification, 6.1% with grade 2 and, finally, 6.1% with grade 3).

However, glenohumeral OA is a common complication also after anatomic shoulder stabilization procedures, such as arthroscopic Bankart repair. A systematic review [22] demonstrated postoperative OA in 26% of shoulders that underwent arthroscopic Bankart repair with suture anchors with a minimum 5 years of follow-up. Considering the risk of developing shoulder OA in both open Latarjet procedure and arthroscopic Bankart repair [25], we support the theory of other authors [20, 24, 52] who consider dislocation arthropathy to be a part of the natural history of shoulder dislocation.

Moreover, a recent meta-analysis [56] even showed that Latarjet procedure provides a lower degree of OA compared to the other treatments, both operative and non-operative.

Risk factors influencing the development or progression of dislocation arthropathy after Latarjet procedure were evaluated with a pooled analysis.

In this analysis, the only statistically significant factor associated with the development of shoulder OA is the overhanging position of the bone graft, as already reported by other authors [2, 18, 43, 45]. For this reason, the surgeons’ experience and proper training for the open Latarjet procedure are necessary to guarantee an appropriate position of the bone graft and minimize the risk of overhanging. Other risk factors, such as the young age at dislocation, the number of dislocations before surgery, and the practice of sport before and after surgery, indicated by several authors [2, 11, 13, 20, 24, 36, 43] as responsible for the development of shoulder OA, have not been confirmed in this study.

On the other hand, the results of the present study show that hyperlaxity is a protective factor for dislocation arthropathy. Lädermann et al. [36] described similar results, supposing this protection should be given by decreasing postoperative contact pressure between the humeral head and glenoid surface.

The principal strength of this study is the study design. The pooled analysis has allowed analysing many patients from different studies who underwent the open Latarjet technique. The availability of raw data has also permitted the comparison of the information among four different studies to minimize data interpretation errors.

Minor variations in surgical technique introduced by surgeons, different lengths of follow-up, and some missing data in the study population represent the limitations of this study. Additionally, all of the included studies were retrospective.

## Conclusion

The Latarjet procedure is a valid and safe surgical treatment in recurrent anterior shoulder instability with a low risk of developing moderate or severe OA also at long-term follow-up. The overhanging position of the bone graft represents the principal risk factor of joint degeneration, whereas the hyperlaxity seems to be protective.

Finally, age, gender, time between first dislocation and surgery, and number of dislocations do not seem to affect the onset of OA after Latarjet procedure.

Therefore, an accurate execution of the Latarjet procedure can be considered a valid treatment even in young and athletes thanks to the low recurrence rates and the low development of major long-term complications.

## Supplementary Information

Below is the link to the electronic supplementary material.

Supplementary file1 (DOCX 13 KB)

## Abbreviations

AP: Anteroposterior
HR: Hazard ratios
MINORS: Methodological Index for Nonrandomized Studies
n.s.: Non-significant
OA: Osteoarthritis
PRISMA: Preferred Reporting Items for Systematic Reviews and Meta-Analyses
95% CI: 95% Confidence intervals
