# Supplementary material for: Low grade of osteoarthritis development after Latarjet procedure with a minimum 5 years of follow-up: a systematic review and pooled analysis
Source: Knee Surg Sports Traumatol Arthrosc. 2021 Oct 22;30(6):2074–83. doi: 10.1007/s00167-021-06771-w (PMC9165270; doi:10.1007/s00167-021-06771-w)
Supplement: Supplementary file 1 — Supplementary file1 (DOCX 13 KB) [file 167_2021_6771_MOESM1_ESM.docx]

Appendix A

**Exact Pubmed/Medline search string up to February 29, 2020**

20|COMBINE: #19 AND #7|54

19|COMBINE: #18 OR #17|326365

18|(arthrosis[TIAB]) OR (degenerative+changes[TIAB])|16928

17|COMBINE: #16 OR #5 OR #11|313488

16|COMBINE: #15 OR #14|176

15|(dislocation[TIAB]) AND (arthropathy[TIAB])|176

14|(dislocation+arthropathy[TIAB])|26

13|COMBINE: #12 AND #7|31

12|COMBINE: #11 OR #5|313372

11|(arthritis[TIAB])|178471

10|COMBINE: #9 OR #5|268726

9|(glenohumeral[TIAB])|6378

8|COMBINE: #5 OR #6|263217

7|COMBINE: #1 OR #2 OR #3 OR #4|1038

6|(glenohumeral+arthritis[TIAB])|346

5|"Arthritis"[MH]|263074

4|(coracoid[TIAB]) AND (bone[TIAB]) AND (block[TIAB])|89

3|(coracoid+bone+block[TIAB])|40

2|(latarjet[TIAB])|606

1|(latarjet[ALL])|1030

**Exact Embase search string up to February 29, 2020**

-------------------------------------

(('arthritis':ab,ti OR 'dislocation arthropathy':ab,ti OR 'degenerative changes':ab,ti OR 'arthrosis':af) AND (('latarjet':ab,ti OR 'coracoid bone block':ab,ti) OR 'latarjet procedure'/exp OR 'latarjet operation'/exp)) AND [embase]/lim NOT ([embase]/lim AND [medline]/lim)

-------------------------------------
